# Does nociception monitor-guided anesthesia affect opioid consumption? A systematic review of randomized controlled trials

**DOI:** 10.1007/s10877-019-00362-4

**Published:** 2019-07-20

**Authors:** Fleur S. Meijer, Marieke Niesters, Monique van Velzen, Chris H. Martini, Erik Olofsen, Ruth Edry, Daniel I. Sessler, Eveline L. A. van Dorp, Albert Dahan, Martijn Boon

**Affiliations:** 1 Department of Anesthesiology, Leiden University Medical Center, Albinusdreef 2 (Postal Zone H5-Q), 2333 ZA Leiden, The Netherlands; 2 Department of Anesthesiology, Rambam Medical Centre, The Ruth and Bruce Rappaport Faculty of Medicine, Technion Institute of Technology, Haifa, Israel; 3 Department of Outcomes Research, Anesthesiology Institute, Cleveland Clinic, Cleveland, OH USA

**Keywords:** Nociception monitoring, Opioid consumption, Systematic review

## Abstract

Monitors that estimate nociception during anesthesia may be used to guide opioid and other analgesics administration to optimize anesthesia care and possibly outcome. We reviewed the literature to evaluate current evidence of the effect of nociception-guided management over standard anesthesia practice during surgery. A systematic review of the literature on the effect of nociception monitoring on anesthesia practice was conducted. Reports were eligible if they compared nociception-guided anesthesia to standard practice during surgery. Primary endpoint of this review is intraoperative opioid consumption. Secondary endpoints included hemodynamic control, postoperative pain and pain treatment. We identified 12 randomized controlled trials that compared one of five different nociception monitoring techniques to standard anesthesia care. Most studies were single center studies of small sample size. Six studies reported intraoperative opioid consumption as primary outcome. There was considerable variability with respect to surgical procedure and anesthesia technique. For nociception monitors that were investigated by more than one study, analysis of the pooled data was performed. The surgical plethysmographic index was the only monitor for which an intra operative opioid sparing effect was found. For the other monitors, either no effect was detected, or pooled analysis could not be performed due to paucity of study data. On secondary outcomes, no consistent effect of nociception-guided anesthesia could be established. Although some nociception monitors show promising results, no definitive conclusions regarding the effect of nociception monitoring on intraoperative opioid consumption or other anesthesia related outcome can be drawn.

*Clinical trial number* PROSPERO ID 102913.

**Electronic supplementary material:**

The online version of this article (10.1007/s10877-019-00362-4) contains supplementary material, which is available to authorized users.

## Introduction

General anesthesia is intended to produce a state of unconsciousness combined with suppression of nociception, allowing the patient to undergo invasive surgical procedures without undue harm or awareness. Nociception is defined by the International Association for the Study of Pain as *the neural process of encoding noxious stimuli*, causing autonomic and/or behavioral responses such as elevation of blood pressure or motor withdrawal reflexes; noxious stimuli are actually or potentially tissue damaging events that occur during surgery [1]. Nociception is generally suppressed by administration of potent opioid analgesics.

Clinicians usually estimate nociception by evaluating hemodynamic responses, along with lacrimation, sweating, increase in pupil diameter or movement. Recently, nociception monitors have been introduced to track nociception during anesthesia and guide administration of analgesics, usually opioids. Since inadequate opioid administration is associated with unwanted hemodynamic responses (e.g. hypertension and hypotension), reliable nociception monitors may help optimize anesthetic management. Evidence from preclinical validation studies show that these monitors distinguish noxious and non-noxious events far better than hemodynamic responses [2–6].

Various nociception monitors are already available, and others are being developed. All use algorithms to assess various physiological variables and they produce numerical indexes that give an estimation of the nociception–antinociception balance (see Table 1). The value of nociception monitors is increasingly assessed in clinical settings, where they are compared to standard anesthesia care. We intended to evaluate current evidence of the effect of nociception-guided management on intraoperative opioid consumption and other anesthesia related outcomes. To this end, a systematic search was conducted to identify reports that assessed intraoperative nociception monitoring versus routine anesthetic management on intraoperative opioid consumption. If possible, analysis of pooled data was performed to synthesize current evidence.

**Table 1:** Individual nociception monitor characteristics

Monitor	Manufacturer	Autonomic input variables	Index	Optimal range/cut off
SPI	GE Healthcare	Pulse beat interval Pulse wave amplitude	0–100	20–50
ANI	MDoloris Medical Systems	Heart rate variability	0–100	50
NOL	Medasense	Heart rate and heart rate variability Pulse wave amplitude Skin conductance	0–100	10–25
CARDEAN	Alpha-2	Blood pressure Heart rate	0–100	60
Algiscan	ID Med	Pupil diameter	NA	Pupil diameter increase > 30%

*SPI* surgical plethysmographic index, *ANI* analgesia nociception index, *NOL* nociception level index, *NA* not available

## Materials and methods

Our goal was to determine whether the use of a monitor or algorithm that estimates patients’ nociceptive state during general anesthesia alters anesthesia management with respect to the administration of opioids. Secondary aims were to evaluate the effect of nociception monitoring on hemodynamic parameters, time related variables (e.g. time to extubation) and postoperative pain score and opioid consumption. We searched for trials in which the primary endpoint was the amount of opioid analgesic medication given during anesthesia. All available nociception monitors were considered, along with indices derived from the electroencephalogram or evoked potentials. The systematic review was conducted in accordance with the PRISMA statements. A planned meta-analysis was registered at PROSPERO (www.crd.york.ac.uk/prospero) under Identifier 102913. Since there was so much heterogeneity among the included studies, a valid meta-analysis could not be conducted. Instead, pooled data were analyzed exclusively within unique monitors, when available.

### Identification of relevant studies

On June 30, 2018 we searched the PubMed electronic database from inception for studies on nociception monitoring. The search strategy is given in Supplemental Document 1; no language or date restrictions were applied. To reduce the risk of missing relevant studies, we checked relevant review papers and a previous meta-analysis [7]. The title and abstracts of the retrieved studies were next step-wise evaluated for the following three criteria: (1) study in surgical patients, aged 18 years and older, (2) study performed during general anesthesia, and (3) randomized trial of nociception monitor-guided administration of opioids versus standard clinical care in which analgesics were administered solely based on blood pressure and heart rate values. All papers meeting all criteria were read in full. Three reviewers (FM, AD, and RE) independently performed the selection procedure.

### Data extraction

The identified reports were searched for the following variables and these were extracted for the review if available: authors, country of origin, year of publication, number of subjects in each treatment group, type of opioid and anesthetic used, opioid consumption during surgery, anesthetic consumption during surgery, duration of anesthesia, hypotension and/or bradycardia events, hypertension and/or tachycardia events, time from end-of-anesthesia or administration of reversal agent until extubation or emergence, duration of stay in the post-anesthesia care unit (PACU), average pain level in the PACU, and opioid consumption in the PACU.

Opioid consumption during surgery was transformed to morphine dose (in mg kg^−1^ h^−1^) using the following conversion ratios: 1 mg morphine (intravenous) = 0.5 mg oxycodone = 10 μg fentanyl = 1 μg sufentanil = 10 μg remifentanil = 50 μg alfentanil. These conversion rates are arbitrarily based on existing opioid potency data [8].

### Bias assessment

Study quality was evaluated with the Cochrane Collaboration’s tool for assessing risk of bias in randomized trials [9]. This tool considers six domains of bias: (1) selection bias which includes the presence of random sequence generation and allocation concealment, (2) performance bias which includes the blinding of participants and personnel, (3) detection bias which includes the blinding of outcome assessment, (4) attrition bias, which includes incomplete outcome data, (5) reporting bias which includes selective reporting, and (6) other bias. For each study, the presence of bias in each domain was assessed independently by two reviewers (FM and MN). Discrepancies in judgment were resolved by consensus and, when required a third reviewer was consulted (AD).

### Data analysis

Analyses of combined data per unique monitor were conducted using the statistical package R (version 3.5.0) with the metafor package [10, 11]. Data were analyzed using random effects models, assuming two sources of variance, within-study error and between-study error. Heterogeneity was by measuring the degree of inconsistency in the studies’ results (I^2^).

## Results

### Study selection

**Fig. 1:**
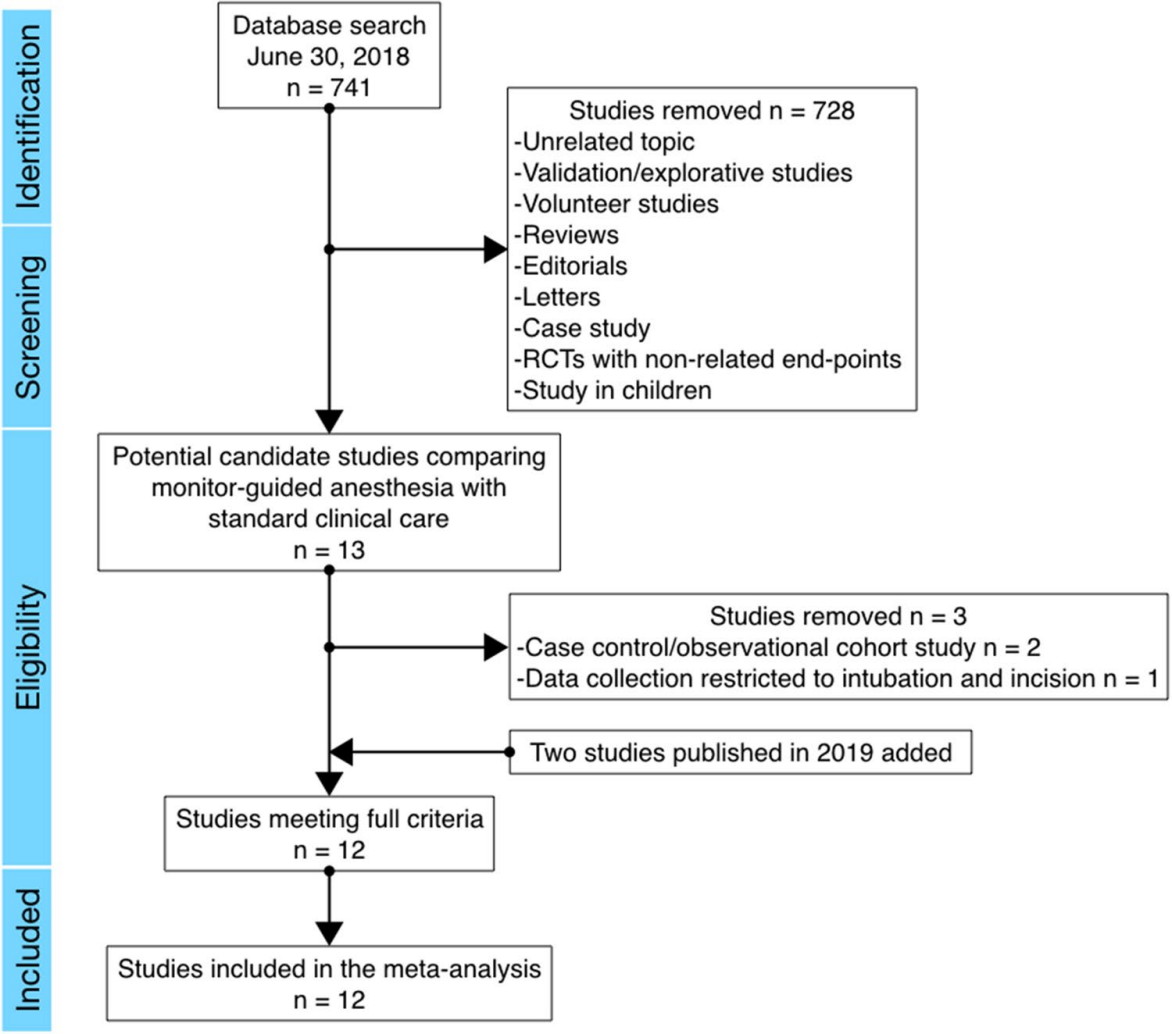
Flow diagram of the study selection process following the PubMed search on June 30, 2018

The flow chart of the PubMed search is shown in Fig. 1, which illustrates retrieval of 741 records. After removal of 728 irrelevant studies, 13 trials were carefully examined and assessed for eligibility. Three papers were removed, one study using the analgesia nociception index (ANI), the other two the surgical plethysmographic index (SPI, Fig. 1) [12–14]. Two of these studies were observational (using a historic control group) or case–control studies [13, 14]; the third study did not report data for the complete duration of the surgical procedure [12]. Finally, two recently published studies were added. The first is a randomized trial from our research group that compared nociception level (NOL)-guided analgesia with standard clinical care in patients undergoing major abdominal surgery [15]. The second study is a trial that compared SPI guided anesthesia with standard care during laparoscopic cholecystectomy [16]. Our review process therefore resulted in a total of 12 unique studies eligible for inclusion in the review.

### Study characteristics

Characteristics of the 12 included studies are shown in Table 2 and the main findings are summarized in Table 3. All publications were in English. A total of 1045 patients were studied, with 520 receiving an intervention compared to 526 treated according to routine clinical care. Care guided using the SPI was evaluated in six studies [16–21] followed by the ANI in three studies [22–24]. The NOL, pupillometry and the beat-to-beat cardiovascular depth of anesthesia index (CARDEAN 2.0) were evaluated in single studies [15, 25, 26]. Next, a discussion of the available evidence for each separate monitor is presented.

**Table 2:** Properties of the included studies in this review

First author	Year	Nociception monitor (target for intervention group^a^)	Number of patients (intervention/control)	Surgery	Anesthesia technique	Hypnosis monitor (target for all patients)	Patients (ASA, age)	Primary outcome	Comments
Won [21]	2016	SPI (20–50)	23/22	Thyroid resection	– Sevoflurane – Oxycodone – Rocuronium	BIS (40–60)	ASA 1–2, 20–65 years.	Intraoperative oxycodone consumption	Single center study. Rescue medication (thiopental) in case of somatic movement despite SPI and BIS values within target boundaries
Bergmann [17]	2013	SPI (20–50 and Δ < 10)	76/75	Orthopedic surgery in a supine or beach-chair position	– Propofol – Remifentanil – Mivacurium	SE (40–60)	ASA 1–3, 18–75 years	Propofol (1) and remifentanil (2) consumption. Recovery time (3)	Single center study. There was maximum remifentanil infusion rate. No difference in postoperative pain
Chen [18]	2010	SPI^b^ (20–50)	40/40	ENT surgery	– Propofol TCI – Remifentanil TCI – Rocuronium	BIS (40–60)	ASA 1–2, 18–70 years	Number of episodes with inadequate anesthesia^d^	Single center study. No difference in postoperative pain
Colombo [19]	2015	SPI (< 50)	30/30	Laparoscopic chole-cystectomy	– Propofol TCI – Remifentanil TCI – *Cis*-atracurium	SE (40–60)	ASA 1–2, 18–50 years	Sympathetic modulation	Single center study
Gruenewald [20]	2014	SPI (< 50)	42/40	Gynecological or orthopedic surgery	– Sevoflurane – Sufentanil – Rocuronium	BIS (40–60)	ASA 1–2, 18–65 years	Incidence of unwanted somatic events	Single center study
Jain [16]	2019	SPI (20–50)	70/70	Laparoscopic cholecystectomy	– Sevoflurane – Fentanyl	BIS (40–60)	ASA 1–2, 18–65 years	Intraoperative fentanyl consumption	Single center study
Dundar [22]	2018	ANI (50–70)	22/22	Breast surgery	– Sevoflurane – Remifentanil – Thoracic para-vertebral block	BIS (40–60)	ASA 1–2, females, 18–65 years	Total remifentanil consumption (mentioned in “Discussion”)	Study sites not specified. All patients received a thoracic paravertebral block
Upton [24]	2017	ANI (≥ 50)	24/26	Lumbar discectomy or laminectomy	– Sevoflurane – Fentanyl and non-opioid analgesia – Rocuronium	BIS (40–60)	ASA 1–2, 18–75 years	Postoperative NRS	Single center study
Szental [23]	2015	ANI (> 50)	59/60	Laparoscopic cholecystectomy	– Sevoflurane or desflurane –Fentanyl, morphine and non-opioid analgesia –Neuromuscular blocker	BIS depth not specified	ASA –, 18–75 years	VAS > 50 mm in first postoperative hour	Study performed in two centers
Meijer [15]	2018	Nociception level (10–25)	40/40	Major abdominal surgery: urology (48%), abdominal surgery (39%), gynecology (14%)	– Propofol TCI – Remifentanil TCI – Rocuronium	BIS (45–55)	ASA 1–3, 18–80 years	Propofol (1) and remifentanil (2) consumption. Incidence of inadequate anesthesia events (3)	Single center study
Martinez [25]	2010	Beat-to-beat cardiovascular index^c^ (< 60 and absence of conventional signs of nociception)	71/75	Gastroscopy and/or colonoscopy	– Propofol TCI – Alfentanil	BIS (40–60)	ASA 1–2, 20–75 years	Reduction in unwanted movements	Single center study. Beat-to-beat blood pressure was measured using the Finapress™ device using a finger cuff.
Sabourdin [26]	2017	Pupillometry (pupil diameter 5–30% of baseline)	25/30	Gynecological surgery	– Propofol TCI – Remifentanil TCI – Atracurium	BIS (40–60)	ASA 1–2, females, 18–60 years	Remifentanil consumption	Single center study. In case of large hemodynamic changes remifentanil TCI could be changed and/or vasoactive medication or fluids could be given

*ANI* analgesia nociception index, *BIS* bispectral index, *ENT* ear-nose-and-throat, *SE* state entropy, *SPI* surgical plethysmographic index

^a^The target indicates adequate analgesia. A monitor value outside the target range leads to additional opioid administration according to the protocol of the individual studies

^b^The authors named their monitoring index the surgical stress index which is equivalent to the SPI

^c^The beat-to-beat cardiovascular depth of anesthesia index (CARDEAN 2.0) is a nociceptive algorithm based on changes in blood pressure and tachycardia

^d^Inadequate anesthesia: mean blood pressure > 120% of baseline or > 100 mmHg *or* mean blood pressure < 80% of baseline or < 60 mmHg; heart rate > 90 beats min^−1^*or* < 80% of baseline or < 45 beats min^−1^; somatic arousal; somatic response

**Table 3:** Summary of outcomes reported by each study

Author	Monitor	Effect on opioid consumption during anesthesia	Absolute difference in opioid consumption (converted to morphine equivalents)	Hemodynamic variables	Efficiency variables (i.e. time to extubation)	Postoperative pain and opioid consumption	Other
Won [21]	SPI	30% Reduction in oxycodone consumption in the SPI group (p = 0.012)	2.8 mg	No significant differences	Extubation time 3.4 min shorter with SPI (p = 0.03)	No significant differences	
Bergmann [17]	SPI	25% Reduction in remifentanil consumption in SPI group (p < 0.05)	0.12 mg kg^−1^ h^−1^	No differences in mean HD variables (apart from intubation: Δ MAP 9 mmHg, p < 0.005)	Recovery times were reduced by 3–4 min in the SPI group (p < 0.05)	No significant differences	
Chen [18]	SPI	23% Reduction in remifentanil consumption in the SPI group (p < 0.05)	0.3 mg kg^−1^ h^−1^	No significant difference in mean values Number of episodes with hypertension, hypotension or bradycardia significantly reduced (p < 0.01)	No significant differences	No significant differences	
Colombo [19]	SPI	No significant differences	NA	There was less sympathetic modulation as measured by heart rate variability indices in the SPI group (p < 0.01)	No significant differences	No significant differences	
Gruenewald [20]	SPI	No significant differences	NA	No differences in mean values or number of episodes	No significant differences	No significant differences	
Jain [16]	SPI	12% Increase in fentanyl consumption in the SPI group (p = 0.017)	0.024 mg kg^−1^ h^−1^	No significant differences	Duration of surgery was 9.8 min reduced in SPI group (p = 0.03)	Fentanyl consumption was 7 μg less in SPI group (p = 0.01) and VAS was 0.6 points lower in SPI group (p = 0.04)	
Dundar [22]	ANI	30% Reduction in remifentanil consumption in the ANI group (p = 0.027)	33.5 mg	No significant differences	No significant differences	No significant differences	
Upton [24]	ANI	No significant differences	No significant differences	No significant differences (only total surgery time reported)	No significant differences	Pain scores in the first 90 min of PACU stay were on average 1.3 units lower pain scores in the ANI group (p = 0.01)	
Szental [23]	ANI	No significant differences	No significant differences	No significant differences	No significant differences	No significant differences	
Meijer [15]	NOL	28% Reduction in remifentanil consumption in the NOL group (p < 0.001)	0.18 mg kg^−1^ h^−1^	No significant differences	Reversal to extubation time was 2 min shorter in NOL group (p = 0.03)	No significant differences	
Martinez [25]	CARDEAN	No significant difference in alfentanil consumption (dose corrected for duration) Increased number of patients in the CARDEAN group received alfentanil (83% vs. 61%; p = 0.003)	NA	No significant differences	No significant differences	No significant differences	There was a 50% reduction in unwanted movements with CARDEAN monitoring at BIS values < 60 (p = 0.001)
Sabourdin [26]	Pupillometry	48% Reduction in remifentanil consumption in pupillometry group (p < 0.001)	0.42 mg kg^−1^ h^−1^	More patients required nicardipine in pupillometry group (42.3% vs. 0%; p < 0.001)	No significant differences	Reduced morphine consumption 0–12 h (mean difference 0.1 mg kg^−1^) No difference in pain scores	Significant correlation between remifentanil consumption and postoperative morphine requirements

Intraoperative opioid consumption was converted to morphine equivalents in mg kg^−1^ h^−1^ if possible

*ANI* analgesia nociception index, *BIS* bispectral index, *CARDEAN* cardiovascular depth of anesthesia index, *ENT* ear-nose-and-throat, *HD* hemodynamic, *NA* not available, *NOL* nociception level, *SE* state entropy, *SPI* surgical plethysmographic index

#### Surgical plethysmographic index (SPI)

Six reports were identified that compared SPI guided anesthesia to standard practice [16–21]. Studies were diverse with regard to the maintenance hypnotic (propofol or sevoflurane) and opioids (remifentanil/sufentanil/fentanyl and oxycodone). The guidance of these agents was however uniform: all maintained a bispectral index or state entropy value between 40 and 60 and steered their SPI values in the intervention group to maintain values below 50. Bergman et al. and Chen et al. found that SPI monitoring reduced remifentanil consumption during ear-, nose- and throat surgery and orthopedic surgery by 23% and 25% respectively [17, 18]. Similarly, Won et al. found a 30% reduction in oxycodone consumption in SPI guided patients for thyroid surgery [21]. However, in absolute values, the reduction in oxycodone from that study equaled to only 2.8 mg morphine. In contrast with these studies, Jain et al. found an increased consumption of fentanyl in SPI guided patients during laparoscopic cholecystectomy [16]. This translated to less postoperative pain and a reduced need for postoperative adjuvant analgesia. All other studies failed to find an effect of SPI guidance on postoperative pain or opioid consumption (see Table 2). In general, the effect of SPI guidance on secondary endpoints was limited. No significant differences in mean hemodynamic values were reported, with the exception of Chen et al., who found that the number of episodes with inadequate anesthesia (a composite endpoint that includes hyper/hypotension and brady/tachycardia) was reduced by 80% under SPI guidance [18]. Recovery times were identical or at best 4 min faster with SPI guidance. Finally, the studies of Colombo et al. [19] and Gruenewald et al. [20] found no differences on intraoperative opioid consumption or perioperative secondary outcomes at all.

*Pooled data* analysis shows that the SPI had an overall significant opioid sparing effect: mean difference in morphine equivalents − 0.06 mg kg^−1^ h^−1^ (95% CI − 0.12 to − 0.00, Z = − 2.0, p = 0.04, I^2^ = 70%), or an 8% reduction in intra operative opioid consumption.

*In conclusion*, analysis of the pooled data showed that SPI guided management may reduce opioid consumption during surgery, although individual study results varied considerably. Heterogeneity was substantial due to differences in methodology, including type of surgery and choice of opioid and hypnotic agents. Therefore, no definitive conclusions can be drawn.

#### Analgesia nociception index (ANI)

Three reports were identified comparing the ANI to standard of care during lumbar discectomy [24], breast surgery [22] and laparoscopic cholecystectomy [23] (see Table 2). All studies used a volatile hypnotic for maintenance (sevoflurane or desflurane). Fentanyl, remifentanil and morphine were used for analgesia. Dundar et al. provided pre-operative single shot thoracic paravertebral blockade for 44 patients receiving breast surgery under general anesthesia [22]. This study found a significant reduction of intra operative remifentanil consumption in the ANI guided group (30% or 33.5 mg morphine equivalents in total, p = 0.027). This significant difference in remifentanil consumption did not translate into faster recovery times or improved pain scores in the PACU. In addition, methodological flaws (for instance, the report fails to detail data collection and blinding procedures) reduce the quality of evidence of this study. The studies of Szental et al. and Upton et al. found no differences in opioid use during anesthesia, however opioid consumption was not the primary outcome of both studies and the use of morphine for intraoperative analgesia may not have been an ideal choice [23, 24]. Regarding other endpoints, only Upton et al., found lower pain scores in ANI guided patients after lumbar discectomy or laminectomy (mean difference first 90 min postoperative 1.3 NRS points, p = 0.01; see Table 2) [24]. However total fentanyl dose in the PACU was not significantly different.

*Pooled data* analysis of these studies show that ANI guidance did not result in a significant difference in intraoperative opioid consumption: mean difference + 0.00 mg kg^−1^ h^−1^ morphine (95% CI − 0.018 to 0.024, Z = 0.12, p = 0.90, I^2^ = 98%).

*In conclusion*, analysis of the pooled data did not show a benefit of ANI guidance on intraoperative opioid consumption. Preliminary effects of ANI monitoring concerning an opioid sparing effect during breast surgery in patients that receive additional neuraxial blockade *or* on postoperative pain scores after back surgery, need to be corroborated in future studies.

#### Nociception level index (NOL)

Our systematic review did not find any study that compared NOL guided anesthesia versus standard care on intraoperative opioid consumption. However, our group recently published a trial in which NOL guided anesthesia was compared to standard care in 80 patients during major ambulatory laparoscopic and open abdominal surgery without the use of neuraxial blockade [15]. General anaesthesia was maintained with propofol (bispectral index target 40–60) and remifentanil (NOL target 10–25 for the intervention group; blinded for standard care group). Propofol and remifentanil were administered using target controlled infusion. This study found a reduction in remifentanil consumption of 28% (absolute reduction 0.18 mg kg^−1^ h^−1^ morphine equivalents; p < 0.001). Additionally this study found a trend towards improved hemodynamic stability. Postoperative pain scores or opioid consumption did not differ significantly (see Table 2).

*In conclusion*, data from only one study indicates that NOL guided anesthesia may reduce intraoperative remifentanil consumption. Future studies are needed.

#### Cardiovascular depth of analgesia (CARDEAN 2.0)

The systematic search yielded one study that assessed the use of the CARDEAN 2.0 monitor versus standard care during procedural sedation for endoscopic procedures [27]. Sedation was administered with the use of target-controlled infusion propofol, aimed at a BIS of 60. Additionally, in the CARDEAN group, alfentanil could be administered when the monitor value exceeded 60. In the standard care, alfentanil could only be administered if a mandatory propofol intervention failed to achieve stability. Due to the nature of this protocol, the CARDEAN group received more doses of alfentanil, although the normalized dose (corrected for procedure time) was not significantly different. The increased use of alfentanil in the CARDEAN group resulted in significantly less unwanted movements during the procedures (50% reduction, p = 0.001), but also a tendency to increased apnea [27].

*In conclusion*, limited evidence from a single center study shows an increase in opioid administration and a reduction in unwanted movements when the CARDEAN is used for procedural sedation. The possibility that the increase in opioid administration is related to the study protocol itself cannot be ruled out.

#### Pupillometry

Pupillometry versus standard care was assessed in one study in 55 patients during major gynaecological surgery [26]. In both groups, anaesthesia was maintained with propofol, aimed at a BIS of 40–60. In the pupillometry group, remifentanil was dosed according to predefined changes in pupil diameter. This study found reduced remifentanil consumption in pupillometry guided patients (mean difference 0.42 mg kg^−1^ h^−1^ morphine equivalents, p < 0.001). This translated to less morphine requirement in the first 12 h after surgery. Pain scores did not differ significantly. In addition, persistent pain was less frequent after 3 months post-surgery in pupillometry guided patients (51% in standard group vs. 13% in pupillometry group, p = 0.004). The reduced intraoperative remifentanil administration in pupillometry group resulted in more administration of nicardipine for hypertensive episodes (see Table 2) [26].

*In conclusion*, data from one study indicates that nociception monitoring by pupillometry may help to reduce opioid consumption during major gynaecological surgery, with possible secondary benefits on short term (less opioid consumption) and midterm (less persistent pain). Future studies are however needed.

### Risk of bias

**Fig. 2:**
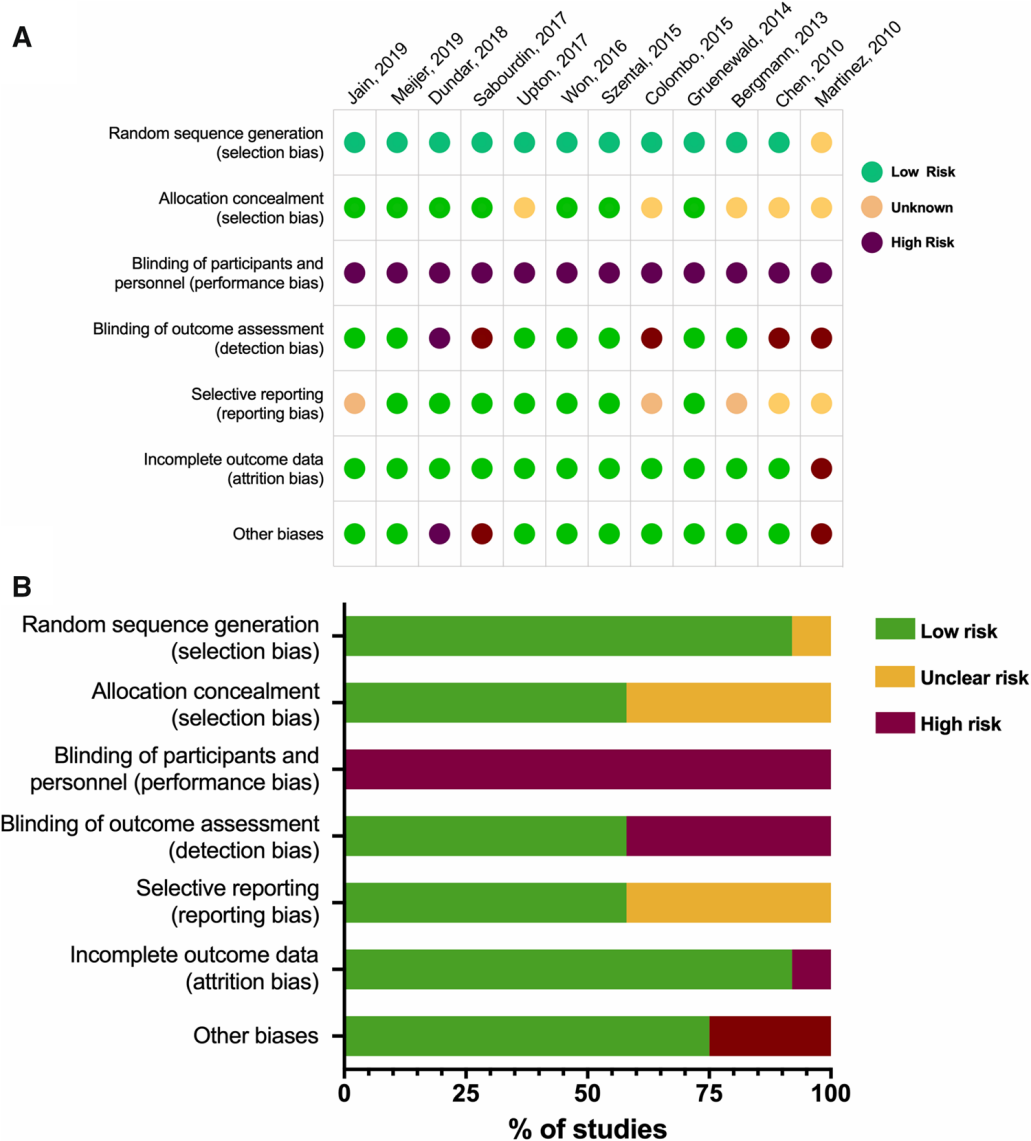
Evaluation of bias in the selected 12 studies. **a** Risk of bias per study according to the domains defined in the Cochrane Handbook for Systematic Reviews of Interventions version 5.1.0 (http://handbook-5-1.cochrane.org). **b** Summary of bias per domain

Risk of bias per study is shown in Fig. 2a and summarized in Fig. 2b. All studies were troubled by the inability to fully blind the investigators due to the nature of the intervention, i.e. need to either use or not-use the monitor during the surgical procedure. Setting aside this inevitable performance risk, seven studies had no additional high risk of bias domain according to the definitions of the Cochrane Handbook for Systematic Reviews of Interventions [15–17, 20, 21, 23, 24], two studies had one additional high risk of bias [18, 19]; and the remaining three studies had two or three additional high risks of bias domains [22, 25, 26]. The most common high risk of bias, apart from the performance bias, was detection bias (five studies) [18, 19, 22, 25, 26] and other bias (three studies) [22, 25, 26].

## Discussion

Our systematic search on the effect of nociception monitoring versus standard care during general anesthesia on opioid consumption yielded 12 reports. Four did not find a significant difference in opioid consumption, seven found a reduced opioid consumption—with widely varying magnitude and one study found an increased opioid consumption (see Table 3). We did not conduct a planned meta-analysis because the data were so heterogeneous. However, a meta-analysis has been published before by Gruenewald et al., despite substantial heterogeneity. They found no significant effect of nociception monitoring on intraoperative opioid consumption or other secondary outcomes, apart from a reduced rate of unwanted movement [7].

We performed sub-analyses of pooled data from unique monitors when more than one trial was available which was the case for the SPI and ANI monitors. These analyses found no repeatable significant effect for the ANI monitor but an opioid sparing effect for the SPI monitor of about 8% or 0.06 mg kg^−1^ h^−1^ morphine equivalents. It is debatable whether this reduction is clinically relevant. Data from single studies concerning the NOL and pupillometry monitors also show a more substantial reduction in intraoperative opioid consumption (0.18 and 0.42 mg kg^−1^ h^−1^ morphine equivalents respectively) [15, 26]. However, these results require confirmation. On secondary outcomes, no consistent beneficial effect of nociception guided management was observed, although admittedly, some individual well conducted trials show promising effects on intraoperative hemodynamic stability [15] or postoperative pain [16, 24, 26] (see Table 2). Again, these results need to be confirmed.

Measurement of nociception under general anesthesia is challenging task. In general, noxious stimuli that are perceived by the autonomic nerve system will evoke autonomic and behavioral responses. The magnitude of these responses depends on the intensity of the stimulus and the presence of any alleviating agents; i.e. the nociception–anti-nociception balance. During anesthesia, autonomic responses are noted by their effects on haemodynamic and respiratory control. Nociception monitors invariably use one or more of these autonomic variables as input for their algorithm to produce an index of nociception (see Table 3). Unfortunately, these autonomic variables are not uniquely related to nociception; any stressing or alleviating factor may cause a change in blood pressure or heart rate. In addition, both the choice of maintenance hypnotic and opioid and the type of surgical procedure profoundly affect the nociception–anti-nociception balance. All these factors may reduce the specificity of nociception monitors when they are tested in the clinical setting.

Patients show large inter-individual differences in their response to noxious stimuli and analgesic therapy. Nociception monitors can be used as an aid to improve individualized antinociceptive therapy in daily practice, however they should not be used to maintain nociception index values within a specific range at all costs. In addition, although some trials showed a reduced opioid consumption in nociception guided patients, the use of these monitors can also result in an increased consumption of opioids or other analgesics for certain procedures [16]. Both ways may improve outcomes for patients, such as reduced postoperative pain. The key utility of these monitors is not reducing opioid consumption per se, rather to achieve the optimal dosing of any analgesic technique for the individual patient that will result in the best outcome.

### General limitations

Current data are troubled by the large heterogeneity that was present among studies. Studies differed significantly in design, study population and surgery and anesthesia type (Table 1). The most commonly performed type of surgery was abdominal surgery (five studies, some of which were laparoscopic) [15, 16, 19, 20, 23, 26]; other procedures included ear-nose-and-throat surgery [18], breast surgery [22], thyroidectomy [21], lumbar discectomy/laminectomy [24], and orthopedic surgery (arthroscopy of knee, shoulder or ankle) [17]. Anesthesia technique varied considerably among studies: six studies used propofol for maintenance of anesthesia [15, 17–19, 25, 26], five combined with remifentanil and one combined with alfentanil. The other studies used a volatile anesthetic (sevoflurane or desflurane) combined with either remifentanil (*n* = 1), fentanyl (*n* = 2), oxycodone (*n* = 1) or sufentanil (*n* = 1) [16, 20–24]. Most studies used a neuromuscular blocker (*n* = 9) [15–21, 23, 24, 26], and one study used a thoracic paravertebral block in both intervention and standard care groups [22]. The ample differences in type of anesthesia and in the intensity of the surgical trauma may profoundly affect the individual study results. For instance, noxious stimuli during gastroscopy under propofol/alfentanil anesthesia will differ significantly in intensity from stimuli during discectomy under sevoflurane/fentanyl anesthesia or breast surgery under sevoflurane/fentanyl surgery with a thoracic paravertebral local anesthetic block [22, 24].

Also, the comparator arm in most trials (commonly defined as *standard clinical care*) often lacked strict guidelines for opioid administration. Therefore, any effect of nociception monitoring on opioid administration could be confounded by suboptimal clinical practice of the comparator group. Finally, all studies included in our review were relatively small with fewer than 100 subjects per treatment arm, and most had fewer than 50 per arm. Additionally, all studies had a high risk of performance bias and five studies had a high risk of detection bias (Fig. 2).

### Limitations of this review

Although our search was extensive, it was limited to the PubMed database and we may have missed some relevant studies. We therefore performed a secondary search across websites of anesthesia societies to detect studies that are presented as abstracts. One possible relevant abstract was detected. Gruenewald et al. performed a multi-center, single blinded randomized-controlled trial in 494 patients studying the influence of SPI and entropy monitoring (i.e. combined analgesia and hypnosis monitoring) versus standard monitoring on signs of unwanted anesthesia events [28]. However, in their preliminary report the authors do not mention opioid consumption, which is the endpoint of this review. No other potentially relevant reports were found. Finally, we only included studies on adult patients, and we did not report on nociception monitoring in children.

## Conclusions

Current data are inconclusive about the effect of nociception monitoring on intraoperative opioid consumption or anesthesia-related outcome. Future homogeneous (randomized and open) and predefined (to reduce heterogeneity and detection risk) trials are needed to improve current level of evidence.

## Electronic supplementary material

Below is the link to the electronic supplementary material.

Supplementary material 1 (DOCX 12 kb)
