# Supplementary material for: Does nociception monitor-guided anesthesia affect opioid consumption? A systematic review of randomized controlled trials
Source: J Clin Monit Comput. 2019 Jul 20;34(4):629–41. doi: 10.1007/s10877-019-00362-4 (PMC7367908; doi:10.1007/s10877-019-00362-4)
Supplement: Supplementary file 1 — Supplementary material 1 (DOCX 12 kb) [file 10877_2019_362_MOESM1_ESM.docx]

**Supplemental document 1:** PubMed search strategy

(((("Analgesia nociception index"[tw] OR "Nociception Level"[tw] OR "noxious stimulation response index"[tw] OR "nociceptive withdrawal reflex"[tw] OR "nociceptive withdrawal reflexes"[tw] OR "nociceptive flexion reflex"[tw] OR "nociceptive flexion reflexes"[tw] OR "nociceptive flexor reflex"[tw] OR "nociceptive flexor reflexes"[tw] OR "nociceptive flexor response"[tw] OR "nociceptive flexor responses"[tw] OR "Nociceptive flexion reflex threshold"[tw] OR "nociceptive flexion reflex thresholds"[tw] OR "nociceptive stimuli"[tw] OR "nociceptive stimulus"[tw] OR "pleth index"[tw])

AND

("surgery"[Subheading] OR "surgery"[tw] OR "surgical procedures, operative"[mesh] OR "general surgery"[mesh] OR "Anesthesia and Analgesia"[Mesh] OR "post-operative"[tw] OR "Postoperative Period"[Mesh] OR "postoperative"[tw] OR "operative"[tw] OR surgical*[tw] OR analges*[ti] OR anesth*[ti] OR anaesth*[ti] OR postanalges*[ti] OR postanesth*[ti] OR postanaesth*[ti]))

OR

(("Nociception"[Mesh] OR "Nociception"[tw] OR Nociception*[tw] OR "pain intensity"[ti])

AND

("monitoring"[tw] OR "Pain Measurement"[Mesh] OR "measurement"[tw] OR "measurements"[tw] OR "measure"[tw] OR measur*[tw] OR assessment*[tw] OR "Numerical Rating Scales"[tw] OR "Verbal Rating Scales"[tw] OR "Visual Analogue Scales"[tw] OR "Numerical Rating Scale"[tw] OR "Verbal Rating Scale"[tw] OR "Visual Analogue Scale"[tw] OR "Visual Analog Scales"[tw] OR "Visual Analog Scale"[tw] OR "Skin conductance level"[tw] OR "Pupillary reflex measurement"[tw] OR "Pupillary reflex measurements"[tw] OR "Pupillary diameter measurement"[tw] OR "pupillary diameter measurements"[tw] OR "pupillary diameter monitoring"[tw] OR "pupillary diameter recordings"[tw] OR "Heart rate variability"[tw] OR "Neuroendocrine response"[tw] OR "Neuroendocrine responses"[tw] OR "Neuro endocrine response"[tw] OR "Neuro endocrine responses"[tw] OR "Stress hormone plasma levels"[tw] OR "Stress hormone plasma level"[tw] OR "Autonomic control"[tw] OR "Haemodynamic variables"[tw] OR "Haemodynamic variable"[tw] OR "Hemodynamic variables"[tw] OR "Hemodynamic variable"[tw] OR "Composite variability index"[tw] OR "Surgical Pleth index"[tw] OR "Surgical Stress Index"[tw] OR "Noxious stimulation response index"[tw] OR "Somatic response"[tw] OR "somatic responses"[tw] OR "somatic responsive"[tw] OR "somatic responsiveness"[tw] OR "somatic responsivity"[tw] OR "Spectral entropy"[tw] OR "Spectral entropies"[tw] OR "State entropy"[tw] OR "Response entropy"[tw] OR "Photoplethysmography"[Mesh] OR "Photoplethysmography"[tw] OR Photoplethysmograph*[tw] OR "Light Reflection Rheography"[tw] OR "Photoreflexometry"[tw] OR Photoreflexometr*[tw] OR "Motor response"[tw] OR "Motor responses"[tw] OR "Skin conductance levels"[tw] OR "Skin conductance level"[tw] OR "rating scale"[tw] OR "rating scales"[tw] OR "pupillary reflex"[tw] OR "pupillary reflexes"[tw] OR "pupillary response"[tw] OR "pupillary responses"[tw] OR "pupillary diameter"[tw] OR "pupillary diameters"[tw] OR "Heart Rate/physiology"[Mesh] OR "Autonomic Nervous System/physiology"[Mesh] OR "Hemodynamics/drug effects"[Mesh] OR "pleth variability index"[tw] OR "pleth index"[tw] OR "Skin conductance"[tw] OR "Pain Measurements"[tw] OR "Pain Assessments"[tw] OR "Pain Assessment"[tw] OR "Analgesia Tests"[tw] OR "Analgesia Test"[tw] OR "Nociception Tests"[tw] OR "Nociception Test"[tw] OR "Pain Questionnaire"[tw] OR "Pain Questionnaires"[tw] OR "Pain Scale"[tw] OR "Pain Scales"[tw] OR "Formalin Test"[tw] OR "Formalin Tests"[tw] OR "Tourniquet Pain Test"[tw] OR "Tourniquet Pain Tests"[tw])

AND

("surgery"[Subheading] OR "surgery"[tw] OR "surgical procedures, operative"[mesh] OR "general surgery"[mesh] OR "Anesthesia and Analgesia"[Mesh] OR "post-operative"[tw] OR "Postoperative Period"[Mesh] OR "postoperative"[tw] OR "operative"[tw] OR surgical*[tw] OR analges*[ti] OR anesth*[ti] OR anaesth*[ti] OR postanalges*[ti] OR postanesth*[ti] OR postanaesth*[ti])))

NOT

("Animals"[mesh] NOT "Humans"[mesh]) NOT ("Electroencephalography"[majr] OR Electroencephalogra*[ti] OR "eeg"[ti] OR "Electromyography"[majr] OR electromyogra*[ti] OR "emg"[ti]))
